# Accurate Instance Segmentation in Pediatric Elbow Radiographs

**DOI:** 10.3390/s21237966

**Published:** 2021-11-29

**Authors:** Dixiao Wei, Qiongshui Wu, Xianpei Wang, Meng Tian, Bowen Li

**Affiliations:** Electronic Information School, Wuhan University, Wuhan 430072, China; weidixiao@whu.edu.cn (D.W.); xpwang@whu.edu.cn (X.W.); mengtian@whu.edu.cn (M.T.); bornlee@whu.edu.cn (B.L.)

**Keywords:** bone extraction, instance segmentation, radiography, convolutional network, pediatric elbow

## Abstract

Radiography is an essential basis for the diagnosis of fractures. For the pediatric elbow joint diagnosis, the doctor needs to diagnose abnormalities based on the location and shape of each bone, which is a great challenge for AI algorithms when interpreting radiographs. Bone instance segmentation is an effective upstream task for automatic radiograph interpretation. Pediatric elbow bone instance segmentation is a process by which each bone is extracted separately from radiography. However, the arbitrary directions and the overlapping of bones pose issues for bone instance segmentation. In this paper, we design a detection-segmentation pipeline to tackle these problems by using rotational bounding boxes to detect bones and proposing a robust segmentation method. The proposed pipeline mainly contains three parts: (i) We use Faster R-CNN-style architecture to detect and locate bones. (ii) We adopt the Oriented Bounding Box (OBB) to improve the localizing accuracy. (iii) We design the Global-Local Fusion Segmentation Network to combine the global and local contexts of the overlapped bones. To verify the effectiveness of our proposal, we conduct experiments on our self-constructed dataset that contains 1274 well-annotated pediatric elbow radiographs. The qualitative and quantitative results indicate that the network significantly improves the performance of bone extraction. Our methodology has good potential for applying deep learning in the radiography’s bone instance segmentation.

## 1. Introduction

Pediatric elbow joint is a complex joint composed of the humerus, ulna, radius, and six age-changing ossification centers [1]. During growth, children have low bone density and mineral content, and may suffer more traumatic factors. Analyzing elbow anteroposterior and lateral radiographs is an effective and straightforward method for a professional orthopedist to diagnose trauma. In the process of pediatric elbow diagnosis, doctors first need to know the locations, shapes, and categories of bones to focus on the abnormal accurately [1]. The ability to accurately distinguish bones depends on the doctor’s professional knowledge and medical experience. However, changes in ossification centers and unossified cartilages make pediatric elbow radiographs more complicated. Emergency physicians who are not familiar with the pediatric elbow joint’s characteristics often encounter pediatric elbow injuries [2]. Overlapping bones in radiographs and vague descriptions sometimes lead to missed diagnosis and misdiagnosis [3]. Data show that fractures of the pediatric elbow represent approximately 12% of systemic fractures [4]. Accurate diagnosis and effective treatment can reduce children’s pain, shorten the healing time, and prevent malunion and neurovascular complications [5].

In recent years, the Deep Convolution Neural Network (DCNN) [6] has developed rapidly and has high precision and stability in medical object location [7,8]. With the help of DCNN, accurately detecting each bone can help doctors diagnose and even assist AI in automatically diagnosing diseases from radiography. Currently, a few studies try to analyze radiographs with DCNN [9,10,11], but all of them treat diagnosis as a normal/abnormal binary classification task. The rough classification task is often only competent for a specific disease and lacks interpretability. However, some elbow injuries usually manifest as bone dislocations such as elbow varus and valgus that need to be diagnosed by judging the relative position between the bones. Without prior knowledge of the position and type of bones, neither doctors nor AI can make a comprehensive diagnosis on a radiograph. Accurate prior knowledge of bones can significantly improve the doctor’s diagnostic accuracy and intelligent interpretation efficiency.

**Figure 1:**
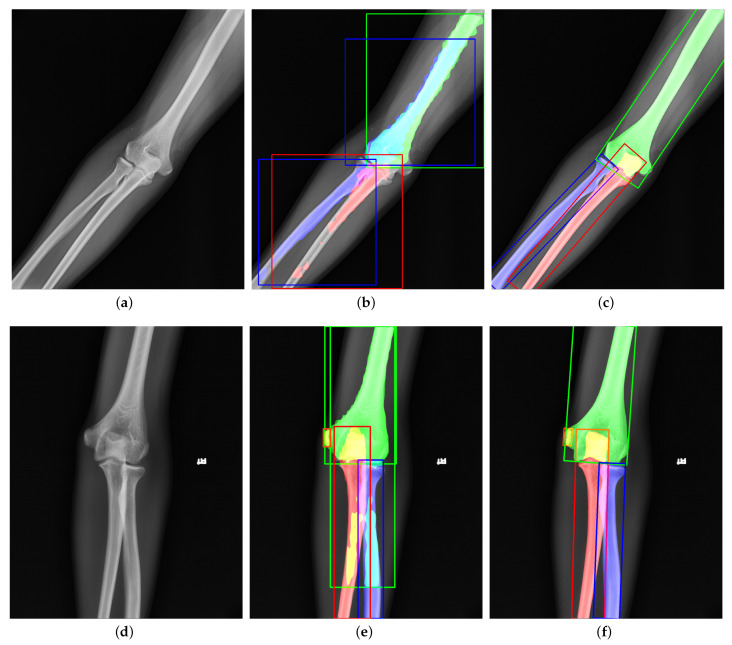
Visualization of (**a**,**d**) original images, (**b**,**e**) outputs of Mask R-CNN [12], and (**c**,**f**) outputs of our proposed method. The green, red, blue, and orange represent the humerus, ulna, radius, and medial epicondyle, respectively. The overlapping regions are denoted by the addition of overlapping bones’ colors. Compared to Mask R-CNN, our results have better performance in bone edges and overlapping areas.

Instance segmentation is a DCNN-based method to generate the pixel-level segmentation mask with the specific category for each target in an image. As far as we know, we are the first to apply instance segmentation on the challenging task of extracting elbow bones. It is natural to take Mask R-CNN [12] as the instance segmentation algorithm. However, Figure 1b,e shows the poor results of edge extraction, bone localization, and bone classification, especially in the overlapping areas. There are three reasons for these results: (i) Mask R-CNN downsamples the original image by many times, and finally outputs a 28×28 binary image as the bone’s segmentation result. Directly upsampling the result to the original image will inevitably lose the bone’s edge information. (ii) Mask R-CNN uses the Horizontal Bounding Box (HBB) to provide a proposal region during pixel-level classification. The horizontal bounding box cannot fit the bones in any direction compactly. As shown in Figure 1b, when the angle between the bone and the horizontal direction is 45${}^{\circ }$, the bounding box probably contains contexts of other bones. The redundant information interferes with the bone segmentation results. (iii) Radiography causes overlapping between bones. Mask R-CNN uses four layers of convolution and upsampling to obtain bone segmentation results. Such a simple structure cannot cope with complex situations such as overlapping bones due to imaging principles. Furthermore, our method solves these problems and provides better results, as shown in Figure 1c,f.

This paper proposes a detection-segmentation network to generate more accurate bone segmentation results in edge and overlapping areas. The previous methods [12,13,14] usually complete object detection and segmentation at the same time. Such methods cannot obtain high-precision bone edges because of direct upsampling. Different from them, we separate object detection and instance segmentation into two steps. We use Faster R-CNN-style architecture to get the approximate position and category of the bones. Then, we use a special segmentation network to classify the bone area at the pixel level; it avoids the loss of information caused by directly upsampling. In the detection stage, inspired by the work in [15] in remote sensing images, we use the Oriented Bounding Box (OBB) instead of the Horizontal Bounding Box (HBB) to wrap the target bone more compactly. The more appropriate bounding box contains less background and redundant information of the adjacent bones, which leads to poor detection and segmentation performance. Different from the remote sensing image, the target in the elbow radiography is more slender. Therefore, we design the special anchor ratio based on the original method to better detect bones. In the segmentation stage, we design the Global-Local Fusion Segmentation Network base on Deeplabv3+ [16] to deal with overlapping areas. Different from Deeplabv3+, our segmentation network adopts a bilateral input method to integrate global information and local information. More rich information reduces the misjudgment of overlapping regions and improves the accuracy of bone edges. The contributions are summarized as follows:

- We design a detection-segmentation architecture to extract each bone from the pediatric elbow radiography.
- We adopt the OBB to clearly describe the bone’s direction and position for enlarging the feature differences between bones.
- We propose the Global-Local Segmentation Fusion Network to fuse the global and local contents of the bone for enhancing segmentation of bone edges and overlapping areas.

## 2. Related Work

**Figure 2:**
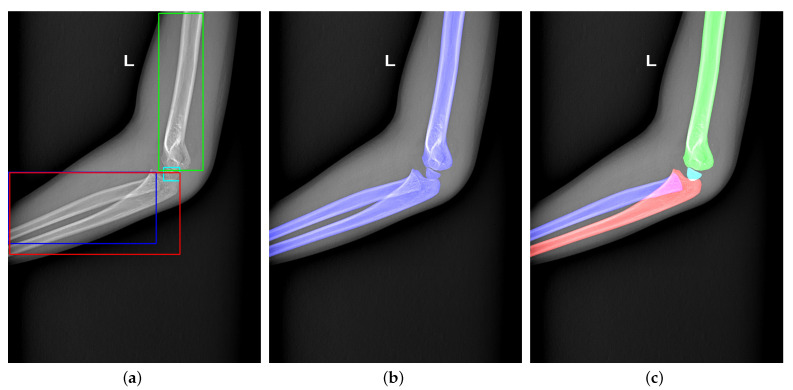
(**a**–**c**) Visualization of object detection, semantic segmentation, and instance segmentation, respectively.

Current DCNN-based object detection, semantic segmentation, and instance segmentation are popular interpretation methods for medical images. Figure 2 shows the three methods’ visualization results, respectively. We will introduce similarities and differences between them as follows.

### 2.1. Object Detection

Object detection is the extension of classification using a rectangular frame to surround the detected target and distinguish its category. Detectors in object detection can be divided into one-stage detectors [17,18,19] and multi-stage detectors [20,21]. One-stage detectors have good running speed but lower accuracy. High-speed detectors are widely used in face detection [22], object tracking [23], etc. [24]. The multi-stage detectors run slower but have higher accuracy. High-precision detectors are often used to detect bones’ approximate location or fractures in radiographs [25]. Guan et al. [26] adjust the structure of Faster R-CNN to detect arm fractures in elbow radiographs. The improved network aims to detect tiny fractures in elbow radiographs. Object detection is also successfully applied on the diagnosis of fractures in wrist radiographs [27], detecting intervertebral discs in lateral lumbar radiographs [28], localizing ossification areas of hand bones [29], and detecting distal radius fractures in anteroposterior arm radiographs [30]. However, object detection can only obtain a rough location of a bone or fracture, which is not enough for further diagnosis. Figure 2a indicates that the rough location is the rectangle’s corners.

### 2.2. Semantic Segmentation

Semantic segmentation can collect more accurate location information from images. This method can classify each pixel in the image as foreground and background to segment the expected object. Common semantic segmentation networks in medicine are Deeplab [16,31,32], U-Net [33], and Unet++ [34]. Badhe et al. [35] implement automated vertebrae segmentation in lateral chest radiographs by U-Net. Zhiqiang Tan [36] design an automatic system to diagnose Adolescent idiopathic scoliosis (AIS) based on the automated spine segmentation. Xie et al. [37] adopt U-Net and Faster R-CNN to detect multiple categories of tuberculosis lesions. First, they use U-Net to segment the lung from chest radiographs. Second, Faster R-CNN is designed to detect multicategory tuberculosis lesions from the segmented lung. The former one aims at reducing unnecessary information and making detect networks focus on the specific tuberculosis area. The latter one classifies multicategory tuberculosis lesions. Using such a cumbersome process to complete the task is done because semantic segmentation cannot identify the same pixel into multiple categories alone. Figure 2b bluntly shows that the semantic segmentation cannot handle multi-classification tasks in overlapping regions.

### 2.3. Instance Segmentation

Combing semantic segmentation and object detection, instance segmentation can detect and segment multiple categories of targets in radiography. The current instance segmentation network mainly adds a Mask branch to the object detection network, such as Mask R-CNN [12], Blend Mask [13], and Hybrid Task Cascade [14]. Instance segmentation is applied to segment lung fields, heart, clavicles, and ribs in chest radiographs [38,39]; pelvis [40]; delineate spinal midline [41]; and to identify unknown bodies by tooth [42].

There are some papers comparing the performance of semantic segmentation and instance segmentation. The work in [41] finds that Mask R-CNN has higher accuracy in pelvis segmentation than U-Net. The work in [43] illustrates that instance segmentation methods are superior to semantic segmentation in tooth segmentation tasks. A vertebrae segmentation comparison experiment shows that instance segmentation performs better than semantic segmentation in vertebrae overlap [44]. Those experiments prove that instance segmentation is more capable of interpreting radiography than semantic segmentation.

Unlike these papers that directly apply the instance segmentation network to complete their tasks, we rebuild the network structure for extracting bones. Our proposal is a more suitable method for bone extraction in elbow radiographs from the perspective of optimizing Mask R-CNN.

## 3. Methodology

**Figure 3:**
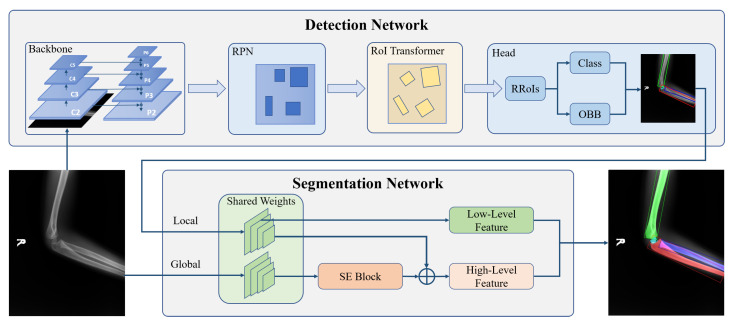
The structure of our proposed bone instance segmentation network. The detection network takes ResNet with FPN as the backbone to generate multi-scales feature maps. The RPN and RoI transformers utilize feature maps to provide rotated regions to predict OBB and classification. Then, each bone in the same image is extracted separately. The segmentation network extracts low-level features and high-level features from the bone and its corresponding original image to generate masks.

Figure 3 shows our proposal structure. To obtain accurate results, we design a detection-segmentation pipeline, which separates instance segmentation into detection and segmentation. The detection network adopts a two-stage detector, which consists of the Backbone network, Region Proposal Network (RPN) [20], RoI Transformer [15], and Head. The Backbone network and RPN are used to extract the bones’ multi-scale features and propose some Regions of Interest (RoIs) where bones may exist in the image. The RoI Transformer aims to predict the bone’s rotation and generate Rotated Regions of Interest (RRoIs). The Head completes two tasks of classification and location. On the other hand, we design the Global-Local Fusion Network for bone segmentation. The segmentation network takes detection results and the original image as input to fuse global and local information for pixel-level classification. The network’s details will be shown as follows. 

### 3.1. Detection Network

#### 3.1.1. Backbone

Pediatric elbow radiographs contain both larger bones such as humerus and smaller ossification centers. The bone instance segmentation task requires both the shallow information for the position and the deep information for classification. Therefore, we adopt a combination of a residual network (ResNet) [45] and a feature pyramid network (FPN) [46] as the backbone. The ResNet ensures that the backbone can extract the deeper bone information without causing network degradation. The FPN fuses shallow and deep information and condenses those into several scales of feature maps. It improves classification and location accuracy and is beneficial to multi-scale bone detection.

The feature pyramid takes the multi-scale outputs $\left\{M_{2},M_{3},M_{4},M_{5}\right\}$ generated by four stages $\left\{C_{2},C_{3},C_{4},C_{5}\right\}$ of ResNet. With a top-down structure, the features in different levels are fused as $\left\{P_{2},P_{3},P_{4},P_{5},P_{6}\right\}$, where $P_{6}$ is the feature 2× downsampled from $P_{5}$ in order to fit a larger scope of the bone. $\left\{P_{2},P_{3},P_{4},P_{5},P_{6}\right\}$ correspond to {4,8,16,32,64} times downsampling size of the original image, which has larger reception fields that are conducive to feature representation.

#### 3.1.2. Region Proposal Network (RPN)

RPN distinguishes positive and negative regions on the feature map and takes a preliminary bounding box regression to generate RoIs. Similar to the original RPN structure [20] based on FPN, we choose anchors with a step size of {4,8,16,32,64} on $\left\{P_{2},P_{3},P_{4},P_{5},P_{6}\right\}$, respectively. Considering that the variability of bone size in pediatric elbow radiographs, we set each anchor with five ratios of {1:4, 1:2, 1:1, 2:1, 4:1}.

#### 3.1.3. RoI Transformer

The OBB commonly develops object detection in remote sensing images [47,48,49]. Compared with the HBB, the OBB can more compactly encapsulate the rotational object, reduce background noise, and obtain extra direction features for object detection. Unlike targets with gravity constraints in nature images, bones in radiographs are arbitrary directionality. On the other hand, an unsuitable bounding box contains too much information about other bones, resulting in the poor performance of pixel-level classification.

**Figure 4:**
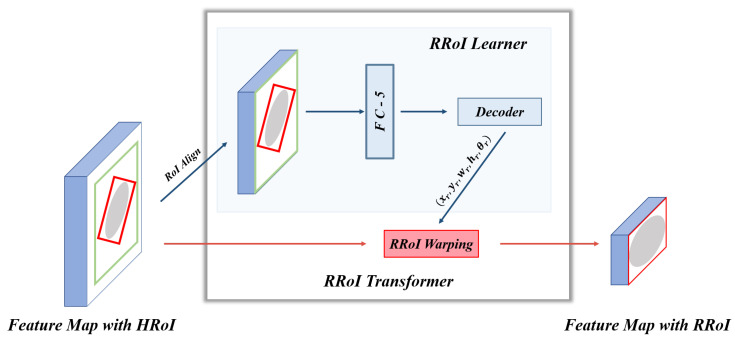
The architecture of RoI Transformer. Each HRoI passes to the RRoI Learner for predicting the target bone’s center points, width, height, and rotation angle. Then RRoI Warping takes RRoI Learner’s output to crop the rotated region from the corresponding feature map. The feature map with RRoI is used for classification and oriented bounding box regression.

**Figure 5:**
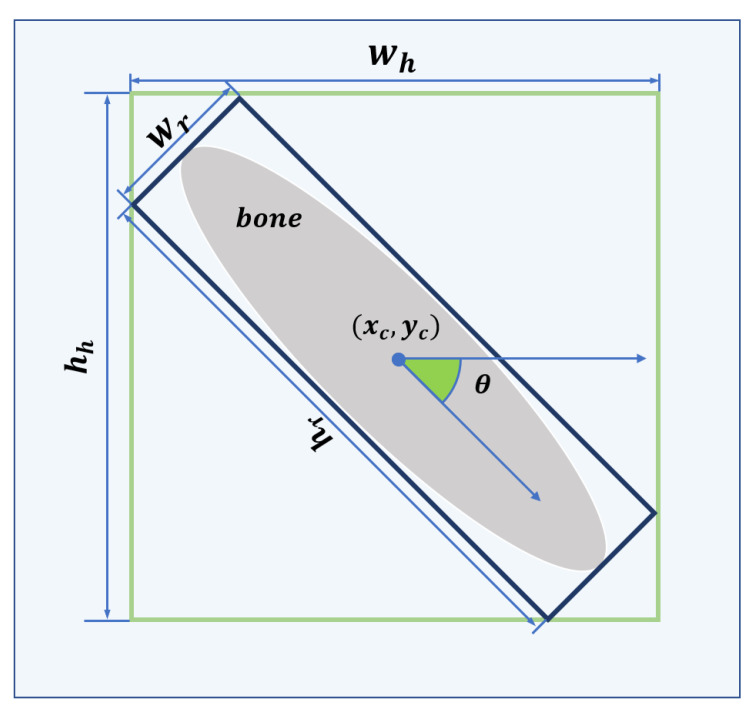
The green and blue bounding boxes are HRoI and RRoI, respectively. The format of the HRoI is $\left(x_{c},y_{c},w_{h},h_{h}\right)$, where $\left(x_{c},y_{c}\right)$ denotes the center of the HRoI and $\left(w_{h},h_{h}\right)$ denotes the width and height of the HRoI. The format of the RRoI is $\left(x_{c},y_{c},w_{r},h_{r},\theta \right)$, where $\left(x_{c},y_{c}\right)$ denotes the center of the RRoI, $w_{r}$ denotes the side of the RRoI parallel to the horizontal axis of the image coordinate system, $h_{r}$ denotes the side of the RRoI parallel to the longitudinal axis of the image coordinate system, and θ denotes the angle between $h_{r}$ and the horizontal axis of the image coordinate system in the range of $\left[-90^{\circ },90^{\circ }\right]$.

For obtaining OBBs, we subjoin RoI Transformer behind the RPN. The RoI Transformer takes Horizontal Regions of Interest (HRoIs) from RPN’s outputs as input and generates RRoIs [15]. As shown in Figure 4, the RoI Transformer consists of RRoI Learner and RRoI Warping. To eliminate possible ambiguity, we cite the definition of RRoI [50]. Figure 5 explains the format of the HRoI and the RRoI.

**RRoI Learner**: The goal of the RRoI Learner is to predict the bone’s angle and scale from HRoIs. After RoI Align, a Fully Connected (FC) layer with the dimension of 5 infers the offsets of Rotated Ground Truths (RGTs) relative to HRoIs. The following equations can calculate the regression targets of offsets relative to RRoIs:

(1) $$\begin{array}{c} t_{x}^{*}=\frac{1}{w_{r}}\left(\left(x^{*}-x_{r}\right)\cos\theta_{r}+\left(y^{*}-y_{r}\right)\sin\theta_{r}\right),\\ t_{y}^{*}=\frac{1}{h_{r}}\left(\left(y^{*}-y_{r}\right)\cos\theta_{r}-\left(x^{*}-x_{r}\right)\sin\theta_{r}\right),\\ t_{w}^{*}=\log\frac{w^{*}}{w_{r}},\;\;\;\;t_{h}^{*}=\log\frac{h^{*}}{h_{r}},\\ t_{\theta }^{*}=\frac{1}{2\pi }\left(\left(\theta^{*}-\theta_{r}\right)\%2\pi \right). \end{array}$$

Here, $\left(x_{r},y_{r},w_{r},h_{r},\theta_{r}\right)$ denotes the center point’s abscissa and ordinate, width, height, and orientation, respectively. $\left(x^{*},y^{*},w^{*},h^{*},\theta^{*}\right)$ is the ground truth of a rotated detection. To avoid confusion, the angle offset target is adjusted in [0,2π] by the mod. The box decoder combines the HRoI and its offset to output the decoded RRoIs.

**RRoI Warping**: RRoI Warping extracts oriented proposal regions with bilinear interpolation from the corresponding feature map and then straightens the extracted regions by Equaiotn (2).

(2) $$\left(\begin{array}{c} x^{\prime }\\ y^{\prime } \end{array}\right)=\left(\begin{array}{cc} \sin\theta & \cos\theta \\ -\cos\theta & \sin\theta \end{array}\right)\left(\begin{array}{c} x-x_{r}\\ y-y_{r} \end{array}\right)+\left(\begin{array}{c} \frac{w_{r}}{2}\\ \frac{h_{r}}{2} \end{array}\right).$$

Here, $\left(x^{\prime },y^{\prime }\right)$ represents the transformed pixel coordinates, $\left(x_{r},y_{r}\right)$ represents the center point coordinates of RRoI in the original image, and $\left(w_{r},h_{r}\right)$ represents the width and height of the RRoI.

#### 3.1.4. Head

After RRoI Warping, all RRoIs are resized to 7×7. Then, a 2048-dimensional FC layer followed by two sibling FCs flatten the features for the classification branch and the oriented bounding box regression branch. The classification branch is used to distinguish the bone’s category. The oriented bounding box regression branch aims at predicting the bone’s center, width, height, and rotational angle.

**Figure 6:**
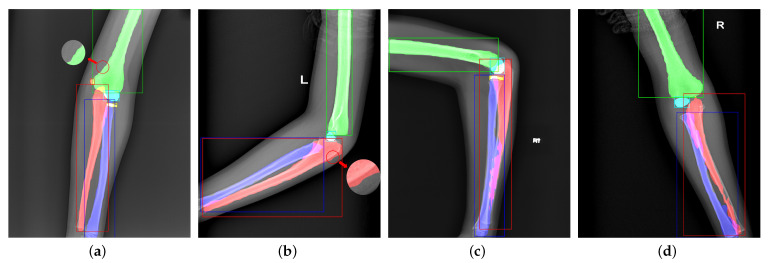
Visualization of failure cases. Panels (**a**,**b**) are caused by too small mask predictions. Panels (**c**,**d**) are due to the unsuitable bounding boxes and weak classification ability of the mask branch.

Unlike Mask R-CNN [12], we do not add a mask branch to the head for the 28×28 mask. Figure 6 shows failure cases from Mask R-CNN. In bounding box representation, we notice that the sizes of ulna, radius, and humerus generally exceed 100×100. However, the sizes of pixel-wise segmentation maps generated by the mask head are 28×28, requiring a 4× upsampling operation to match the original instances. As shown in Figure 6a,b, the contradiction leads to a poor precision in bone boundary extraction, which is critical in radiography interpretation. On the other hand, the mask branch is too simple to handle overlapping bones and bounding boxes. Figure 6c,d shows the segmentation failures that the network cannot distinguish the pixels in overlapping areas.

### 3.2. Global-Local Context Fusion Segmentation

**Figure 7:**
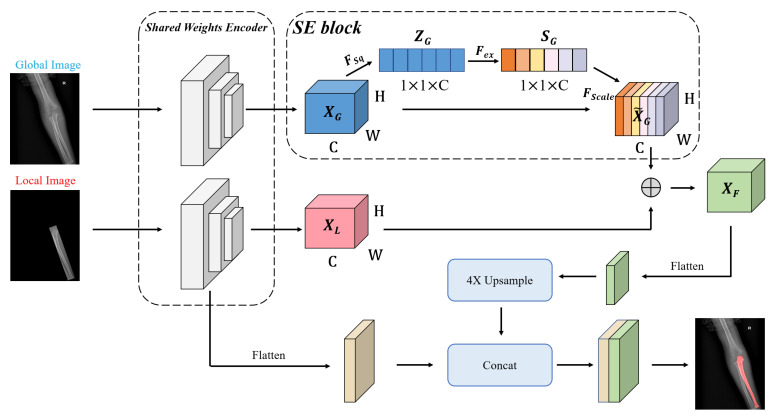
The structure of the segmentation network. The global image is the source image. The local image has the same size as the original image and retains the original image information in the OBB.

Figure 6 shows some failure cases of the overlapping areas. The mask branch in Mask R-CNN can only distinguish whether the pixel is a bone and cannot determine its type. As shown in Figure 7, to enhance the network’s pixel-level resolution, we design the Global-Local Fusion Segmentation Network to generate each bone mask in a semantic segmentation manner. Based on DeepLabv3+ [16], the network adopts an encoder–decoder structure to combine high-level and low-level features to optimize the overlapping areas’ detection results.

**Encoder**: The encoder is designed to extract abundant high-level features. Considering the difficulty of classification in the overlapping areas, we use a bilateral network to combine global and local image information for the segmentation. In detail, two branch networks share weights, and both use Atrous Spatial Pyramid Pooling (ASPP) to generate feature maps $X_{G}$ and $X_{L}$. The ASPP can better capture multi-scale spatial information to adapt to various bones in pediatric elbow joints of different sizes. The benefits of sharing weights are reducing parameters, simplifying the network, and increasing the calculation speed.

**Figure 8:**
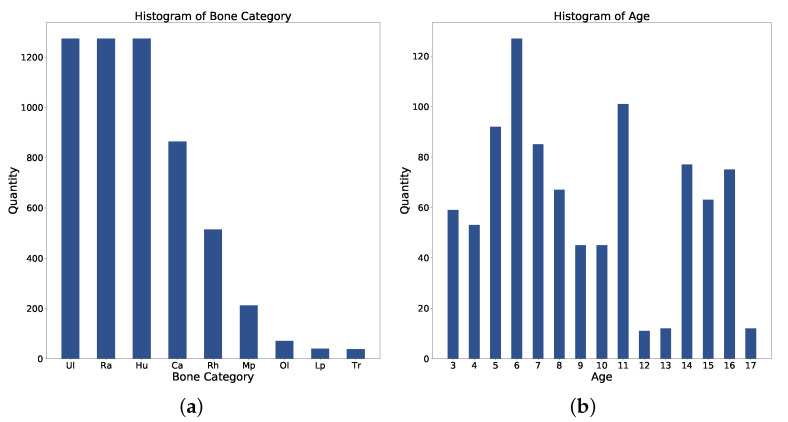
Statistics of (**a**) Bone category distribution. (**b**) Age distribution. Ul: Ulna; Ra: Radius; Hu: Humerus; Ca: Capitellum; Rh: Radial Head; Mp: Medial Epicondyle; Ol: Olecranon; Lp: Lateral Epicondyle; Tr: Trochlea.

The squeeze-and-excitation (SE) block [51] is used to learn an extract weight for each channel of $X_{G}$ in the global branch. The weight of the channel can enhance important features in objective areas and suppress redundant features. As shown in Figure 8, the block $X_{G}$ with the size of W×H×C is squeezed into the feature map $Z_{G}$ with the size of 1×1×C by a Global Average Pooling (GAP). The $Z_{G}$ is calculated by

(3) $$Z_{G}=F_{Sq}\left(X_{G}\right)=\frac{1}{W\times H}\sum_{i=1}^{H}\sum_{j=1}^{W}\alpha (i,j),$$

where $F_{Sq}$ represents the function of GAP and α(i,j) denotes any pixel in $X_{G}$. Two FC layers are used to capture the correlation between feature channels, and then we normalize it by a sigmoid activation:

(4) $$S_{G}=F_{ex}\left({\bar{Z}}_{G}\right)=\mathrm{sigmoid}\left(\delta \left({\bar{Z}}_{G}\right)\right),$$

where sigmoid represents the sigmoid activation, $S_{G}$ represents the obtained weights with the size of 1×1×C, and δ represents the two FC layers.

Then, the weights are merged into the original feature map $X_{G}$:

(5) $${\tilde{X}}_{G}=F_{Scale}\left(S_{G},X_{G}\right)=S_{G}\cdot X_{G}.$$

The SE block stimulates compelling features extracted in the global branch, allowing it to better integrate useful global information to refine features. Finally, we perform element-wise addition on the global and local branches to get a fused feature map $X_{F}$. $X_{F}$ is flattened to the high-level feature by a 1×1 convolution layer.

**Decoder**: The decoder takes both high-level and low-level features as inputs. Thus, it receives much more spatial semantic information. We apply 1×1 convolution on low-level features to reduce the number of channels and bilinearly upsample high-level features by 4× to align the feature shape to perform feature fusion. Then, two features with different semantic information are fused by channel concatenation. Two 3×3 convolutions followed by another 4× bilinear upsampling are used to refine the features and generate a bone mask.

### 3.3. Multi-Task Loss Function

The object detection network is trained by a joint loss function

(6) $$L_{D}=L_{\mathrm{RPN}}+\alpha_{1}L_{\mathrm{cls}}\left(c_{t},{\hat{c}}_{t}\right)+\alpha_{2}L_{\mathrm{reg}}\left(r_{t},{\hat{r}}_{t}\right),$$

where $L_{\mathrm{RPN}}$ represents RPN loss [20], $L_{\mathrm{cls}}$ denotes object classification cross entropy (CE) loss, and $L_{\mathrm{reg}}$ is oriented bounding box regression [15]. We take $\alpha_{1}=1,\alpha_{2}=1$ to configure the head.

The loss function of segmentation network is

(7) $$L_{S}=\alpha_{1}L_{\mathrm{Decode}}+\alpha_{2}L_{\mathrm{Head}},$$

where $L_{\mathrm{Decode}}$ represents the decoder loss, $L_{\mathrm{Head}}$ denotes the FC layers loss [16]. We set $\alpha_{1}=1,\alpha_{2}=0.4$ for segmentation loss weight.

## 4. Experimental Results

### 4.1. Dataset

The dataset contains 1274 pediatric elbow radiographs with scales from 1140×1432 to 1780×1600. Radiographs are screened between January 2003 and October 2010. Among them, 692 radiographs are anterior and 582 are lateral. The whole dataset is separated into training, validation, and test with a ratio of {3:1:1}. As shown in Figure 8a, there are nine bone categories: humerus, radius, ulna, capitellum, radial head, olecranon, trochlea, medial epicondyle, and lateral epicondyle. Figure 8b shows the age distribution. Each bone in this dataset is annotated with an OBB and a mask. Three senior orthopedic specialists cooperate in labeling ground truths with the annotation tool. The tool allows the specialist to wrap any bones with a set of dots in each radiography. The radiographs have been approved by the local ethics committee for this study and we hid the patient’s information before providing it to the investigators.

### 4.2. Implementation Details

Our experiments are implemented with 4 NVIDIA Titan Xp GPUs and Pytorch. The batchsize is set to 16, and the input resolution is 1024×1024. In the object detection network, we use SGD with a weight decay of 0.0001 and momentum of 0.9. The model is trained by 48 epochs with an initial learning rate of 0.02 and it decreases by 10× at epoch 18 and 36. We set the batch size of HRoI, RRoI, and OBB to {256,512,512} per image with ratios {1:1, 1:3, 1:3} of positive to negatives. In the segmentation network, we use SGD with a weight decay of 0.0005 and momentum of 0.9. The model is trained by 8000 iterations with an initial learning rate of 0.01, a minimum learning rate threshold of 0.0001, and it declines by the polynomial decay with a power of 0.9 every epoch. Both the baseline Mask R-CNN and our proposed networks use the same weights for initialization which is pretrained on the ImageNet.

For robustness and the balance of sample orientation characteristics, we resize the original images into the scales of {0.5,1,1.5}. Besides, each training image is randomly rotated within a range of $\left[-90^{\circ },90^{\circ }\right]$ or flipped with a probability of 0.5.

### 4.3. Comparison with Mask R-CNN

To compare the performance of our network and Mask R-CNN, we both use ResNet-50 [45] with FPN as the backbone in Mask R-CNN and our detection network. We use instance-level evaluation, which consists of AP${}_{0.50}$ and AP${}_{0.85}$, to assess network instance segmentation ability. Note that AP${}_{0.85}$, a stricter evaluation standard, is used to evaluate the segmentation effect of medical image instances. As shown in Table 1, for AP${}_{0.50}$, our network is up to 4.7% higher. For AP${}_{0.85}$, it can upgrade the AP from 29% to 45.1%, which shows that our network has a higher segmentation accuracy for bones in radiographs.

**Figure 9:**
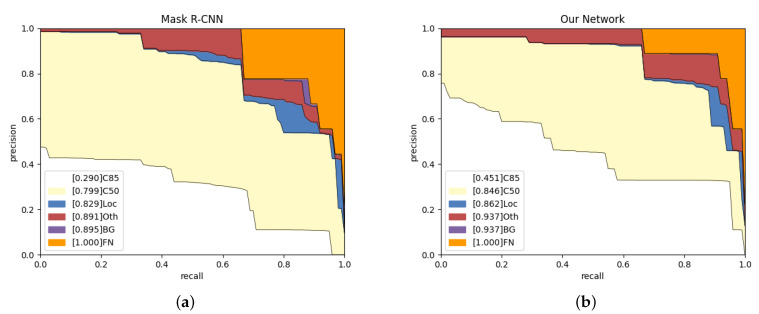
Analysis results on Mask R-CNN and our network in the test set. (**a**,**b**) The evolving proportion of False Positive (FP) types. Loc: deviated position. Oth: classification errors. BG: confusion in background.

To reveal the reasons for false positives, we conduct experiments to observe their distribution and trends in the test set. As shown in Figure 9, we calculate the precision and recall, and then generate the PR curve. We adopt the public object detection evaluation standards from in [52]. The items are as follows.

**C85:** PR curve at IoU = 0.85 corresponds to the area under curve of $AP^{IoU=0.85}$ metric.

**C50:** PR curve at IoU = 0.50 corresponds to the area under curve of $AP^{IoU=0.50}$ metric.

**Loc:** PR curve at IoU = 0.1. The localization errors are ignored. The mask overlaps (IoU∈[0.1,0.5]) with any ground-truth is defined as the localization error.

**Oth:** PR curve after all class confusion is removed. All others objects are assumed to the same class in the question.

**BG:** PR curve after all background (and class confusion) FPs are removed.

**FN:** PR curve after all remaining errors are removed.

According to Figure 9, our network is 16.1% higher than Mask R-CNN on C85, which indicates that our network can obtain more high-quality instance segmentation results. Our network has an increase of 3.3% and 4.6% on Loc and BG, respectively, which shows more robust target recognition and localization capabilities. The blue area represents the proportion of low-precision segmentation results in the network results. Compared with Mask R-CNN, our network has a lower localization error rate. The red area from our network is more extensive than from Mask R-CNN as we obtain the wrong objects, but Mask R-CNN failed to detect them. The purple area is the probability that the network mistakes the background for bones, and the orange area represents the network’s missed detection rate. Purple and orange show that our network has no background classification errors and a lower missed detection rate.

For the large bones of humerus, radius, and ulna, AP${}_{50}$ does not increase significantly, while AP${}_{0.85}$ has a considerable improvement (especially in radius and ulna). Usually, the radius is next to the ulna. Using HBB to warp them in Mask R-CNN will contain too much other bone and ground noise. Redundant noise and the weak ability of pixel-level classification lead to the low precision in AP${}_{0.85}$. Capitellum is a small bone and often overlaps with the humerus, radius, and ulna. Our network gets 11.6% promotion in AP${}_{0.85}$, which explains the fact that the Global-Local Fusion Network has a robust pixel-level classification ability in overlapping areas.

**Figure 10:**
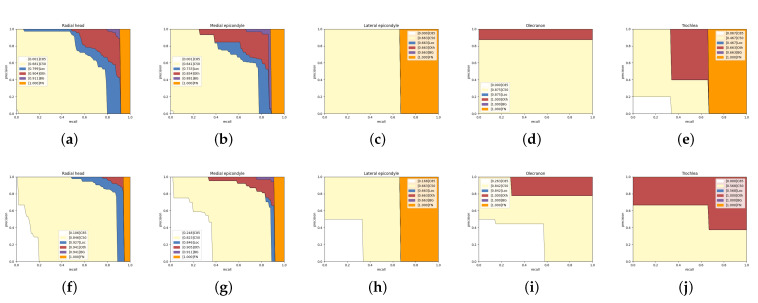
Detailed analysis of Mask R-CNN and our network on five categories in the test set. The first line is the result of Mask R-CNN and the second line is ours. Panels (**a**,**f**): Radial head. Panels (**b**,**g**): Medical epicondyle. Panels (**c**,**h**): Lateral epicondyle. Panels (**d**,**i**): Olecranon. Panels (**e**,**j**): Trochlea.

Figure 10 shows the other five types of bone analysis results. Mask R-CNN’s results in the radial head and medial epicondyle having almost no high-quality segmentation results (AP${}_{0.85}$), and most of the errors come from positioning offset and category confusion. In contrast, our network eliminates most of the mistakes from location and classification, and obtains some high-quality segmentation results. Figure 10c,d indicates that lateral epicondyle and olecranon errors are category confusion, but our network obtains more accurate results. Trochlea does not get a satisfactory improvement on AP${}_{0.85}$. However, we notice that Mask R-CNN do not detect all trochlea, but our network detect them and classify them in the wrong category. The trochlea, lateral epicondyle, and medial epicondyle are age-restricted and can only be discovered in anteroposterior radiographs. Too few training samples lead to poor performance.

### 4.4. Ablation Experiments

We conduct a group of ablation experiments to verify the effectiveness of our combined method. The Mask Head RoI Transformer aims to add a mask head based on the work in [15]. GLFS-Net is the improved network based on Deeplabv3+.

As shown in Table 2, the last four methods perform better than the first two, which explains why our first part is effective. In the last four methods, we evaluate the effectiveness of RoI Transformer and GLFS-Net, respectively. Compared with Faster R-CNN and Deeplabv3+, the RoI Transformer and GLFS-Net have a better performance. Finally, we integrate three parts (RoI Transformer and GLFS-Net) and achieve the best performance among all methods.

### 4.5. Fusion of Traditional Methods and DCNN

Traditional algorithms such as the watershed algorithm [53], superpixel segmentation [54,55], and edge operators can also complete the task of bone segmentation. However, traditional algorithms cannot obtain classification results from the extracted edge information, handling the stacking area, and complete tasks fully automatically. Therefore, we try to combine traditional methods with DCNN to urge the network to pay more attention to the bones’ edges. Therefore, we preprocess the input image to enhance the edge information and observe the network performance. We extract the original image’s boundary with Sobel and cover a channel in the original image. Finally, we used Mask R-CNN to train the preprocessed images and tested the model.

According to Table 3, we find that emphasizing the bone edge cannot significantly improve the network results. Replacing the green or blue channel even impedes the network’s upgrade. We infer that there are two reasons for the degeneration. One explanation is that the edge extraction with Sobel may generate noise among the arms, obstructing the network’s attention to other features of bones. Another reason is replacing the information of a specific channel may destroy the original image balance and continuous information, resulting in the loss of the data. Sometimes artificially inserting some new information into the image and forcing the network to record with prior knowledge may be counterproductive.

### 4.6. Visualization Analysis

**Figure 11:**
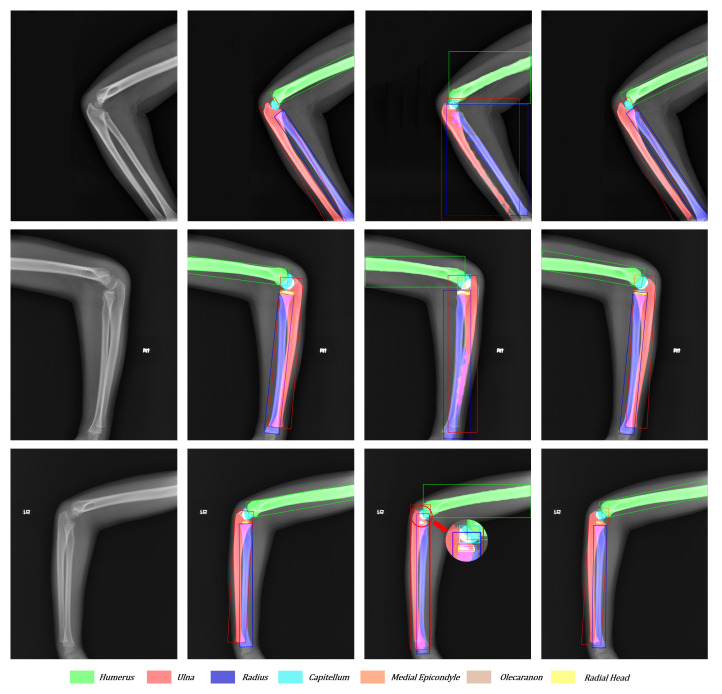
Visualization of the original image, ground truth, Mask R-CNN’s results, and our network’s results from left to right. Each color corresponds to a bone. The overlapping areas are represented as the addition of overlapping bones’ colors.

**Figure 12:**
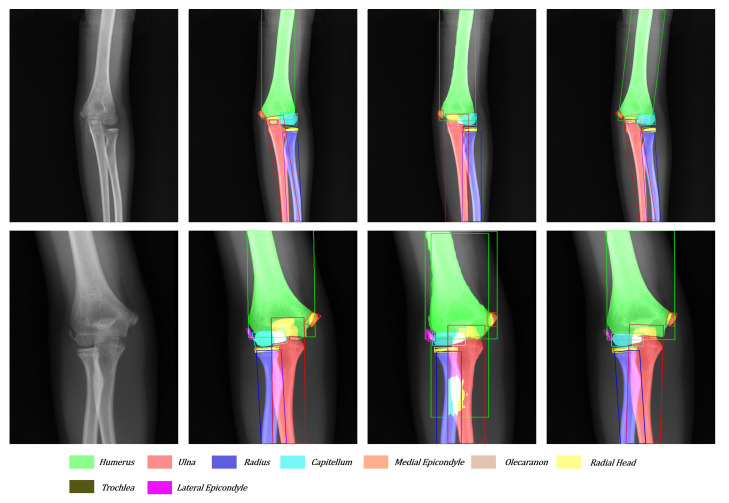
Another visualization of the original image, ground truth, Mask R-CNN’s result, and our network’s result from left to right.

To compare the effect of network improvement, we take the original image, Mask R-CNN, Ground Truth, and our network together in Figure 11 and Figure 12. According to Figure 11, the third column (Mask R-CNN’s results) shows that horizontal bounding boxes of the ulna and radius are often highly coincident, which leads to poor performance in segmentation. However, the fourth column (our network’s results) obtains a higher precision in bone boundary and correct segmentation results because of the more suitable bounding box and the better segmentation methodology. In addition, the third sample illustrates that the large target bone and the small target bone are often close to each other. The superficial mask branch tends to mistake the tiny target bone for the large and overwhelm the tiny target. The Global-Local Fusion Network can correctly distinguish the pixel classification in the overlapping area of bounding boxes based on the target bone’s information and position information relative to other bones. On the other hand, the radial head is repeatedly detected. With the directional characteristics, the OBBs increase the robustness of multi-scale detection and reduce the possibility of retaining multiple bounding boxes of the same classification.

As shown in Figure 12, the tiny target often hides behind the big target in the anteroposterior pediatric elbow radiographs. The trochlea and ulna often overlap entirely. After adding directional features to each bone, our network can easily detect and segment small targets hidden behind large targets. Moreover, facing the various kinds and serious overlap radiography like in the third sample, Mask R-CNN cannot give a satisfactory answer. With the more suitable bounding boxes and more robust segmentation method, our network reaches the level close to the ground truth.

## 5. Conclusions

This article proposes a detection-segmentation network to extract bones from pediatric elbow radiographs. Aiming at the problems of the low edge accuracy and confusion in identifying overlapping regions, we first use the OBB to replace the HBB for describing bones precisely. The OBB can pack the target bones more compactly with the directional feature and find small targets hidden behind large targets. Based on Faster R-CNN, we add an RoI Transformer behind RPN to predict the target’s location, size, and direction. Then, we design a segmentation network called Global-Local Fusion Segmentation Network to solve the overlapping area identification problem. The segmentation network takes the whole image and the local image as a more prosperous basis to distinguish the overlapping bone’s edge and category. The experimental results indicate that our proposal improves the edge accuracy and segmentation ability of overlapping areas.

Although our network aims at pediatric elbow radiographs, each part of our method can be extended to other radiographs (such as knee radiographs) with similar characteristics, which will be further explored in our future work. 

## Figures and Tables

**Table 1:** Quantitative analysis of our proposed method and Mask R-CNN in the test set of our proposed dataset. The best result is highlighted in bold.

Bone Category	Mask R-CNN			Our Network		
	mAP	AP${}_{0.50}$	AP${}_{0.85}$	mAP	AP${}_{0.50}$	AP${}_{0.85}$
All	0.537	0.799	0.290	**0.607**	**0.846**	**0.451**
Humerus	0.879	0.988	0.956	**0.950**	0.985	**0.985**
Radius	0.754	0.970	0.725	**0.890**	**0.980**	**0.945**
Ulna	0.741	0.967	0.688	**0.871**	**0.980**	**0.939**
Capitellum	0.654	0.955	0.288	0.653	0.925	**0.404**
Radial Head	0.324	0.765	0.002	**0.433**	**0.846**	**0.106**
Olecaranon	0.513	0.875	0.050	**0.610**	0.842	**0.263**
Trochlea	0.366	0.467	0.067	0.165	**0.568**	0.000
Medial Epicondyle	0.428	0.641	0.001	**0.508**	**0.823**	**0.248**
Lateral Epicondyle	0.169	0.663	0.000	**0.382**	0.663	**0.168**

**Table 2:** Ablation experiment.

Method	mAP	AP${}_{0.50}$	AP${}_{0.85}$
Mask R-CNN [12]	0.537	0.799	0.290
Mask Head RoI Transformer	0.517	0.812	0.311
Faster R-CNN [20] & Deeplabv3+ [16]	0.556	0.822	0.354
Faster R-CNN & GLFS-Net	0.585	0.832	0.401
RoI Transformer & Deeplabv3+	0.567	0.836	0.389
RoI Transformer & GLFS-Net (ours)	0.607	0.846	0.451

**Table 3:** Quantitative analysis of Mask R-CNN in the test set of three preprocessing methods.

Preprocess Method	mAP	AP${}_{0.50}$	AP${}_{0.85}$
Original images	0.537	0.799	0.290
Replace the red channel	0.472	0.758	0.299
Replace the green channel	0.444	0.718	0.277
Replace the blue channel	0.330	0.609	0.120

## Data Availability

The data used to support the findings of this study are available from the corresponding author upon request. The data are not publicly available due to privacy. We are actively working with hospitals to obtain public permission for the dataset. After obtaining the public permission, we will immediately publish the data on https://github.com/shadowy000/Pediatric-Elbow-Radiography-Dataset.
